# Independent and joint associations of visceral adiposity and insulin resistance with hyperuricemia: A large-scale cross-sectional study

**DOI:** 10.1371/journal.pone.0357105

**Published:** 2026-09-22

**Authors:** Lusha Li, Jingkai Yan, Xixuan Cai, Jianjiang Pan, Liying Chen

**Affiliations:** Department of General Practice, Sir Run Run Shaw Hospital, School of Medicine, Zhejiang University, Hangzhou, Zhejiang, China; University of Diyala College of Medicine, IRAQ

## Abstract

**Background:**

Driven by rapid lifestyle transitions, hyperuricemia (HUA) has evolved into a formidable public health challenge. While insulin resistance (IR) and visceral adiposity are core pathophysiological factors associated with HUA, their relative dominance under discordant metabolic phenotypes remains unclear. We aimed to evaluate the joint and discordant associations of the triglyceride-glucose (TyG) index (surrogate for IR) and lipid accumulation product (LAP) (surrogate for visceral adiposity) with HUA risk.

**Methods:**

This cross-sectional study included 60,516 participants (35,593 males, 24,923 females) from a large medical checkup cohort (January to December 2025). Participants were cross-classified into four metabolic phenotypes based on TyG and LAP medians: Double-low (N = 26,023), Double-high (N = 25,998), Isolated High LAP (N = 4,236), and Isolated High TyG (N = 4,259). Multivariable logistic regression and restricted cubic splines were utilized to assess risk stratifications and non-linear dose-response relationships. Additionally, additive interactions and subgroup analyses were conducted to evaluate synergistic effects and population heterogeneity.

**Results:**

LAP and TyG exhibited independent and additive associations with HUA, exhibiting no significant multiplicative interaction (P for interaction = 0.965) but a significant synergistic effect on the additive scale (Attributable Proportion: 15.1%). Among discordant phenotypes, Isolated High LAP demonstrated a striking dominance over Isolated High TyG in its association with HUA risk. Spline curves revealed that without concurrent high LAP, extreme TyG elevations failed to sustain a high HUA risk, exhibiting a paradoxical decline. Subgroup analysis unmasked crucial demographic heterogeneities: this TyG attenuation was particularly pronounced in males and individuals with a BMI ≥ 24 kg/m². Conversely, the high LAP phenotype conferred the most pronounced relative risk in females (OR: 2.45), effectively abrogating their inherent estrogenic urate-excretory protection.

**Conclusions:**

Visceral adiposity, represented by LAP, emerges as the principal metabolic correlate of HUA. Without the concurrent structural and metabolic burden of visceral fat, IR alone is insufficient to maintain extreme serum uric acid elevations. These findings suggest the clinical importance of prioritizing visceral fat reduction and agents with dual metabolic-uricosuric benefits over isolated glycemic control in HUA management.

## Introduction

In recent years, sweeping changes in lifestyle have led to a dramatic rise in the global prevalence of hyperuricemia (HUA), with a notable trend toward younger demographics [1,2]. Once considered merely a traditional cause of gout, independent risk factor for systemic multiorgan dysfunction, thereby evolving into a formidable public health challenge [3–5]. To mitigate this escalating threat, identifying its upstream metabolic correlates is essential. Currently, insulin resistance (IR) and visceral adiposity are widely acknowledged as the core factors associated with HUA [6], and integrating surrogate markers for both conditions yields substantially superior predictive capacity for risk stratification compared to evaluating them in isolation [7]. However, accurately quantifying these pathophysiological states in large-scale epidemiological settings remains challenging. While conventional anthropometric metrics like body mass index (BMI) are widely utilized, they fail to differentiate body composition and cannot capture the specific lipotoxic burden of visceral adiposity [8]. Consequently, the Lipid Accumulation Product (LAP), a novel composite incorporating waist circumference and triglycerides, has been rigorously validated as a superior, highly specific indicator of visceral fat accumulation and its associated metabolic risks compared to BMI [9,10]. Concurrently, the pathophysiological impact of insulin resistance (IR) on urate metabolism is profound and distinct [11]. Biological studies indicate that hyperinsulinemia actively promotes uric acid reabsorption by upregulating uric acid transporter 1 (URAT1) and glucose transporter 9 (GLUT9) on the proximal tubular epithelial membranes, potentially exerting a synergistic effect with sodium-glucose cotransporter 2 (SGLT2)-mediated solute transport [12,13]. To translate these microscopic mechanisms into population-level risk assessment, the triglyceride-glucose (TyG) index has emerged as a robust and accessible surrogate for IR [14,15].

While the individual associations between LAP, TyG, and HUA have been extensively documented [16], these two pathophysiological states frequently cluster together in metabolic syndrome [17]. To date, large-scale cohorts have rarely cross-classified these indices to evaluate their relative risks under discordant metabolic phenotypes (e.g., isolated visceral adiposity versus isolated insulin resistance). Therefore, when these two core drivers manifest discordantly, it remains unclear which factor serves as the predominant factor associated with HUA. Furthermore, population heterogeneity raises the question of whether this predominant factor shifts across different demographic subgroups [18,19].

In light of these considerations, leveraging a large-scale cross-sectional cohort, this study aims to cross-classify LAP and TyG indices to investigate their joint and discordant associations with hyperuricemia, seeking to identify the primary metabolic correlate and provide precise, phenotype-specific targets for clinical intervention.

## Materials and methods

### Study participants

**Fig 1:**
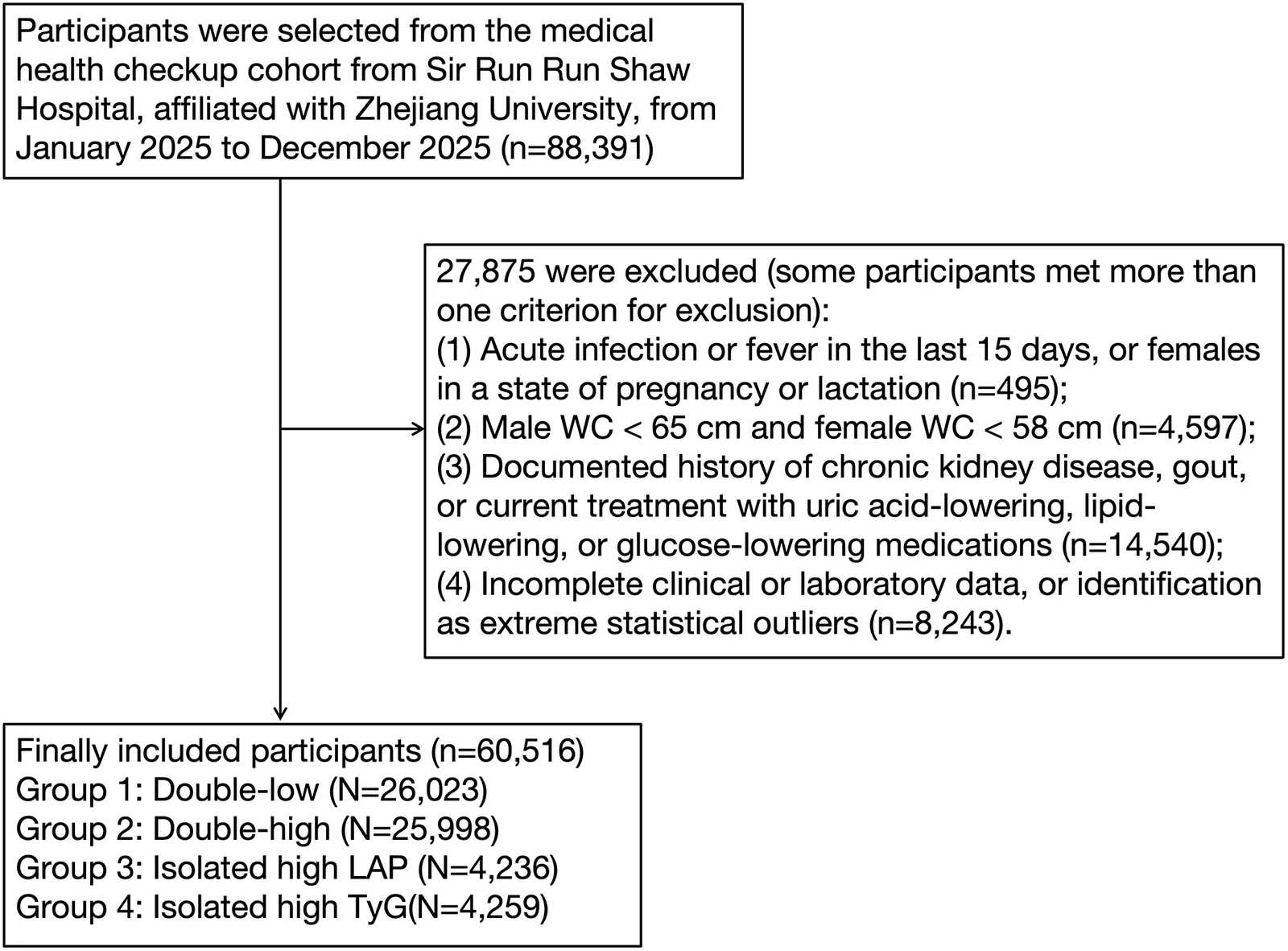
Flowchart of the participant selection process.

Fig 1 illustrates the participant selection process. The initial cohort comprised 88,391 individuals (aged 18–98 years) who underwent routine medical health checkups at Sir Run Run Shaw Hospital, affiliated with Zhejiang University, from January 2025 to December 2025.

Participants meeting any of the following criteria were excluded: (1) Acute infection or fever within the past 15 days, or females currently pregnant or lactating; (2) Males with a waist circumference < 65 cm and females with a waist circumference < 58 cm, as these values yield a zero or negative result in the Lipid Accumulation Product (LAP) calculation [20]; (3) A documented history of chronic kidney disease, gout, or current treatment with uric acid-lowering, lipid-lowering, or glucose-lowering (antidiabetic) medications; and (4) Incomplete clinical or laboratory data, or identification as extreme statistical outliers(e.g., values falling outside the 1st and 99th percentiles for continuous variables such as the TyG index). Following these rigorous exclusions, a final analytical cohort of 60,516 participants was established.

All researchers underwent standardized training in questionnaire administration and data collection methodologies prior to the investigation. This study was approved by the Committee on Human Research at Sir Run Run Shaw Hospital, affiliated with Zhejiang University [Approval No: 2024-2096-01], and was conducted in strict accordance with the Declaration of Helsinki.

### Clinical data collection and anthropometric measurements

The questionnaire encompassed demographic variables and lifestyle factors. Smoking and drinking statuses were defined according to World Health Organization criteria. Specifically, smoking was defined as consuming ≥1 cigarette/day continuously for >6 months (or having quit for less than six months), and drinking was defined as alcohol consumption ≥1 time/week continuously for >6 months (or having abstained for less than six months).

Anthropometric and physiological measurements were performed following standardized protocols. Blood pressure was measured twice using a calibrated mercury sphygmomanometer after a 5-minute rest, and the average was recorded, in accordance with the scientific statement from the American Heart Association (AHA) [21]. Body weight and height were measured with participants wearing light clothing and no shoes. Waist circumference (WC) was measured at the midpoint between the inferior costal margin and the anterior superior iliac spine. These anthropometric procedures and the calculation of Body Mass Index (BMI, weight in kg divided by height in m²) strictly adhered to the standardized guidelines recommended by the World Health Organization (WHO) [22].

### Biochemical analysis

Fasting venous blood samples were collected from all participants to assess essential metabolic profiles. Serum levels of uric acid (SUA), fasting blood glucose (FBG), triglycerides (TG), total cholesterol (TC), low-density lipoprotein cholesterol (LDL-C), and high-density lipoprotein cholesterol (HDL-C) were quantified using an automated biochemical analyzer with compatible reagents, following well-established laboratory assessment protocols widely utilized in recent large-scale metabolic studies [23,24]. Hyperuricemia (HUA) was defined as serum uric acid levels > 420 umol/L in males and > 360 umol/L in females [25,26]. To evaluate the specific impacts of visceral adiposity and insulin resistance, the following surrogate indices were calculated [27,28]. The Lipid Accumulation Product (LAP) was calculated based on sex-specific formulas [29]. The Triglyceride-Glucose (TyG) index was calculated as previously validated [30].

#### Lipid Accumulation Product (LAP).


$$\begin{array}{c} \text{Males}:\text{ LAP}=\text{TG }(\text{mmol}/\text{L})\times (\text{WC }[\text{cm}]-\text{65}) \end{array}$$

$$\begin{array}{c} \text{Females}:\text{ LAP}=\text{TG }(\text{mmol}/\text{L})\times (\text{WC }[\text{cm}]-\text{58}) \end{array}$$

#### Triglyceride-Glucose (TyG) index.


$$\begin{array}{c} \text{TyG}=\text{ln }[\text{TG }(\text{mg}/\text{dL})\times \text{FBG }(\text{mg}/\text{dL})/\text{2}] \end{array}$$

*Note: TG and FBG values measured in mmol/L were converted to mg/dL by multiplying by 88.5 and 18, respectively).*

### Cross-classification of metabolic phenotypes

To isolate and evaluate the independent, joint, and discordant effects of visceral adiposity and insulin resistance on HUA risk, participants were cross-classified into four distinct phenotypic groups based on the median values of the LAP (25.44) and TyG (6.99) indices within the cohort: Group 1 (Double Low: LAP < 25.44 and TyG < 6.99); Group 2 (Double High: LAP ≥ 25.44 and TyG ≥ 6.99); Group 3 (Isolated high LAP: LAP ≥ 25.44 and TyG < 6.99); and Group 4 ((Isolated high TyG: LAP < 25.44 and TyG ≥ 6.99). Given the absence of universally established clinical thresholds for the LAP and TyG indices, this median-based dichotomization was employed to provide a robust 2 × 2 analytical framework, ensuring sufficient statistical power within the discordant subgroups and facilitating a clear clinical interpretation.

### Statistical analysis

Continuous variables were presented as means ± standard deviations (SD) or medians with interquartile ranges (IQR) depending on the data distribution, and were compared using Student’s t-test or the Mann-Whitney U test, respectively. Categorical variables were expressed as frequencies (%) and compared using the Chi-square test.

Multivariable logistic regression models were used to estimate odds ratios (ORs) and 95% confidence intervals (CIs) for the independent associations of the LAP and TyG indices with hyperuricemia (HUA) risk. These models were adjusted for demographic and clinical covariates, including age, gender, smoking, drinking, and blood pressure. Prior to the multivariable logistic regression, collinearity diagnostics were performed to evaluate potential multicollinearity among the covariates. A Variance Inflation Factor (VIF) of less than 5 was considered indicative of no significant multicollinearity [31].

To evaluate the relative risks of discordant metabolic phenotypes, participants were cross-classified into four groups based on the median values of LAP (25.44) and TyG (6.99). Multivariable logistic regression was performed using Group 1 (Double Low) as the reference category. Additionally, a multiplicative interaction term (LAP × TyG) was included in the model to test for the interaction between the two indices. To assess the joint effects from a public health perspective, we evaluated the additive interaction between LAP and TyG. The additive interaction was quantified by calculating the Relative Excess Risk due to Interaction (RERI), the Attributable Proportion due to interaction (AP), and the Synergy Index (S) along with their 95% CIs. A significant positive additive interaction is established if the lower limits of the 95% CIs for RERI and AP are > 0, and S is > 1.

To characterize the non-linear dose-response relationship between the TyG index and the risk of HUA, restricted cubic splines (RCS) were incorporated into the multivariable logistic regression models. The splines were fitted with four knots located at the 5th, 35th, 65th, and 95th percentiles of the TyG index distribution to balance model fit and smoothness. The median TyG value (6.99) was predetermined as the reference point (Odds Ratio = 1.0). The potential non-linearity of the dose-response curve was rigorously evaluated using an analysis of variance (ANOVA) to test the significance of the non-linear terms.

Subgroup analyses were conducted stratified by gender, age (<60 vs. ≥ 60 years), and BMI (<24 vs. ≥ 24 kg/m²). These specific stratifications were determined a priori based on well-established biological mechanisms, such as the distinct sex-dependent physiological distribution of fat and the uricosuric effects of estrogen [32,33]. Additionally, to control the Type I error rate for multiple comparisons in our subgroup analyses, P-values for interaction were adjusted using the Benjamini-Hochberg False Discovery Rate (FDR) method.

All statistical analyses were performed using R (version 4.2.1), and SPSS (version 26.0). A two-tailed *P* < 0.05 was considered statistically significant.

## Results

### Baseline characteristics

A total of 60,516 participants (mean age 44.57 ± 12.46 years; 58.8% males) were included, with an overall hyperuricemia (HUA) prevalence of 30.2%. Baseline clinical and biochemical characteristics across the four cross-classified metabolic phenotypes are presented in Table 1.

**Table 1:** Baseline clinical and laboratory characteristics of the participants according to LAP and TyG joint phenotypes.

Characteristics	Total (N = 60,516)	Double-low (N = 26,023)	Double-high (N = 25,998)	High LAP/ Low TyG (N = 4,236)	Low LAP/ High TyG (N = 4,259)	P-value
Age (years)	44.57 ± 12.46	41.00 ± 11.89	47.65 ± 11.90	47.35 ± 13.19	44.79 ± 12.63	<0.001
Gender, n (%)						<0.001
Male	35,593 (58.8%)	10,423 (40.1%)	19,975 (76.8%)	2,830 (66.8%)	2,365 (55.5%)	
Female	24,923 (41.2%)	15,600 (59.9%)	6,023 (23.2%)	1,406 (33.2%)	1,894 (44.5%)	
BMI (kg/m²)	23.87 ± 3.31	21.90 ± 2.45	25.79 ± 2.89	26.35 ± 2.65	21.67 ± 1.98	<0.001
Waist circumference (cm)	82.73 ± 10.97	75.62 ± 7.95	89.62 ± 8.03	91.80 ± 12.48	75.07 ± 5.95	<0.001
Systolic BP (mmHg)	121.64 ± 16.59	115.62 ± 15.18	127.24 ± 15.96	125.10 ± 15.86	120.82 ± 16.50	<0.001
Diastolic BP (mmHg)	72.83 ± 11.03	68.81 ± 9.97	76.70 ± 10.78	74.45 ± 10.59	72.09 ± 10.28	<0.001
Fasting blood glucose (mmol/L)	5.45 ± 1.09	5.10 ± 0.49	5.79 ± 1.38	5.17 ± 0.48	5.72 ± 1.41	<0.001
HbA1c (%)	5.57 ± 0.69	5.36 ± 0.37	5.78 ± 0.85	5.49 ± 0.38	5.66 ± 0.88	<0.001
Triglycerides (mmol/L)	1.59 ± 1.43	0.84 ± 0.22	2.43 ± 1.84	1.11 ± 0.16	1.48 ± 0.30	<0.001
Total cholesterol (mmol/L)	5.09 ± 0.98	4.81 ± 0.89	5.36 ± 1.01	4.94 ± 0.92	5.22 ± 0.97	<0.001
HDL-C (mmol/L)	1.31 ± 0.30	1.45 ± 0.30	1.16 ± 0.24	1.26 ± 0.26	1.33 ± 0.28	<0.001
LDL-C (mmol/L)	3.29 ± 0.75	3.04 ± 0.67	3.53 ± 0.74	3.25 ± 0.71	3.40 ± 0.73	<0.001
Current smoker, n (%)	13,116 (21.7%)	3,123 (12.0%)	8,254 (31.7%)	999 (23.6%)	740 (17.4%)	<0.001
Alcohol consumption, n (%)	18,764 (31.0%)	5,559 (21.4%)	10,681 (41.1%)	1,443 (34.1%)	1,081 (25.4%)	<0.001
Serum uric acid (μmol/L)	361.53 ± 92.77	319.18 ± 78.41	404.22 ± 89.42	373.32 ± 83.97	347.96 ± 80.51	<0.001
Hyperuricemia, n (%)	18,298 (30.2%)	3,914 (15.0%)	11,958 (46.0%)	1,442 (34.0%)	984 (23.1%)	<0.001

The Double-high group had the poorest cardiometabolic profile and the highest prevalence of HUA (46.0%), while the Double-low group had the most favorable profile and the lowest HUA prevalence (15.0%). Differences between the two discordant groups were also distinct. Compared to the Isolated high TyG group (Low LAP/ High TyG), the Isolated high LAP group (High LAP/ Low TyG) showed higher mean serum uric acid levels (373.32 vs. 347.96 μmol/L) and a higher HUA prevalence (34.0% vs. 23.1%).

### Associations of metabolic phenotypes with HUA

Collinearity diagnostics indicated no severe multicollinearity among the independent variables in the fully adjusted model, with all VIF values well below the threshold of 5 (maximum VIF = 3.409). Multivariable logistic regression analysis was performed to evaluate the associations of metabolic phenotypes with HUA risk (Table 2). In the fully adjusted model (Model 3), the Double-high group showed the highest HUA risk (OR: 2.54, 95% CI: 2.41–2.68, P < 0.001) compared to the Double-low reference group. Among the discordant phenotypes, the Isolated high LAP phenotype (Group 3) was associated with a higher HUA risk (OR: 2.02, 95% CI: 1.87–2.19, P < 0.001) than the Isolated high TyG phenotype (Group 4, OR: 1.25, 95% CI: 1.15–1.36, P < 0.001).

**Table 2:** Multivariable logistic regression analysis for the association between metabolic phenotypes and the risk of hyperuricemia.

Phenotype	Model 1 (Unadjusted)	Model 2 (Adjusted)	Model 3 (Fully Adjusted)
Group 1 (Double-low)	1.00 (Reference)	1.00 (Reference)	1.00 (Reference)
Group 2 (Double-high)	4.81 (4.61–5.02), P < 0.001	3.94 (3.76–4.12), P < 0.001	2.54 (2.41–2.68), P < 0.001
Group 3 (Isolated high LAP)	2.92 (2.71–3.13), P < 0.001	2.54 (2.36–2.74), P < 0.001	2.02 (1.87–2.19), P < 0.001
Group 4 (Isolated high TyG)	1.70 (1.57–1.84), P < 0.001	1.56 (1.44–1.69), P < 0.001	1.25 (1.15–1.36), P < 0.001

Model 1 was unadjusted. Model 2 was adjusted for age, gender, smoking, and drinking. Model 3 was further adjusted for systolic blood pressure (SBP), diastolic blood pressure (DBP), low-density lipoprotein cholesterol (LDL-C), and high-density lipoprotein cholesterol (HDL-C).

There was no significant multiplicative interaction between the LAP and TyG indices (P for interaction = 0.965). However, to explore the joint effects underlying the highest risk observed in Group 2, we evaluated their interaction on an additive scale. This analysis revealed a significant positive additive interaction between high LAP and high TyG on the risk of HUA (RERI = 0.408, 95% CI: 0.229–0.586; AP = 0.151, 95% CI: 0.086–0.216; Synergy Index = 1.316, 95% CI: 1.149–1.507).

### Dose-response relationship stratified by LAP

**Fig 2:**
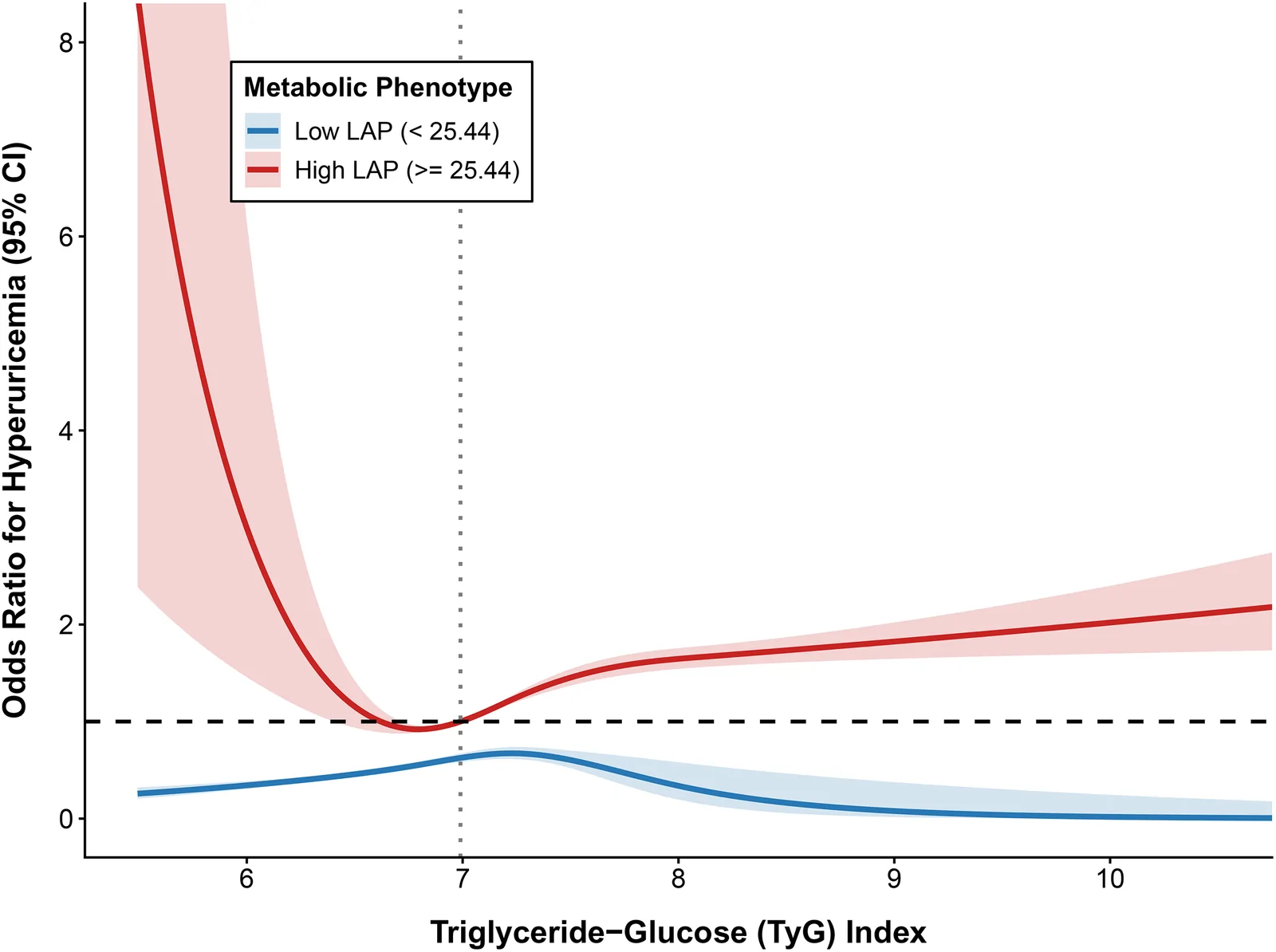
Dose-response relationship between TyG index and hyperuricemia risk, stratified by LAP median.

Restricted cubic spline analysis revealed a significant non-linear dose-response relationship between the TyG index and the probability of HUA (P for non-linearity < 0.001). Smoothing spline curves stratified by the LAP median were plotted to evaluate the dose-response relationship between the TyG index and HUA risk (Fig 2). The high-LAP group maintained a substantially higher risk (Odds Ratio) of HUA across the TyG spectrum compared to the low-LAP group. Within the low-LAP stratum, the risk of HUA initially increased with rising TyG levels but decreased at extreme high TyG levels. In the high-LAP stratum, the risk of HUA remained consistently elevated across high TyG levels.

### Subgroup analysis of discordant phenotypes

As shown in Table 3, subgroup analyses were stratified by gender, age (< 60 vs. ≥ 60 years), and BMI (< 24 vs. ≥ 24 kg/m²). The Isolated high LAP phenotype (Group 3) consistently demonstrated a higher HUA risk than the Isolated high TyG phenotype (Group 4) across all strata. Significant effect modifications were observed for gender and BMI (both P for interaction ≤ 0.001). Notably, these interaction effects remained highly significant (FDR-adjusted P = 0.0003 for gender, and 0.0015 for BMI) even after applying the FDR correction. Conversely, the interaction for age was not significant (P for interaction = 0.419), although the association between Group 4 and HUA lost statistical significance in individuals aged ≥ 60 years (OR: 1.09, 95% CI: 0.85–1.39).

**Table 3:** Subgroup analyses for the associations of discordant metabolic phenotypes with the risk of hyperuricemia.

Subgroup	Group 3 (Isolated high LAP) OR (95% CI)	Group 4 (Isolated high TyG) OR (95% CI)	P for interaction
**Gender**			**< 0.001**
Male	1.77 (1.62–1.94)	1.09 (0.98–1.21)	
Female	2.45 (2.12–2.82)	1.55 (1.34–1.78)	
**Age (years)**			**0.419**
< 60	1.89 (1.75–2.06)	1.22 (1.12–1.33)	
≥ 60	1.68 (1.37–2.05)	1.09 (0.85–1.39)	
**BMI (kg/m²)**			**0.001**
< 24	1.63 (1.36–1.95)	1.38 (1.26–1.52)	
≥ 24	1.51 (1.37–1.66)	1.12 (0.91–1.37)	

Models were adjusted for gender, age, smoking, drinking, systolic blood pressure (SBP), diastolic blood pressure (DBP), low-density lipoprotein cholesterol (LDL-C), and high-density lipoprotein cholesterol (HDL-C).

### Sensitivity analysis

To verify the robustness of our primary findings, a sensitivity analysis was conducted by excluding 703 extreme demographic outliers (individuals aged ≥ 80 years or with a BMI ≥ 35 kg/m²). The associations remained highly consistent. The Isolated high LAP phenotype (Group 3) consistently maintained a substantially higher HUA risk (OR: 2.05, 95% CI: 1.90–2.21) compared to the Isolated high TyG phenotype (Group 4) (OR: 1.28, 95% CI: 1.17–1.39), indicating that our findings were not driven by extreme values (S1 Table).

## Discussion

In this large-scale cross-sectional study involving 60,516 Chinese participants, we decoupled the metabolic phenotypes of visceral adiposity and insulin resistance to elucidate their distinct contributions to hyperuricemia (HUA). We confirmed that both the LAP and TyG indices are independent risk factors for HUA, operating through parallel pathways without a significant multiplicative interaction (P for interaction = 0.965). Our analysis of discordant phenotypes revealed that visceral fat accumulation acts as a primary factor associated with urate dysmetabolism. Specifically, the HUA risk associated with Isolated high LAP (Group 3, OR: 2.02) was significantly greater than that of Isolated high TyG (Group 4, OR: 1.25). These findings are consistent with previous studies by Li et al. [34] and Chen et al. [35], which showed that LAP exhibited greater discriminatory ability for hyperuricemia than the TyG index. Similarly, another study reported that lipid accumulation often exacerbates metabolic disorders even in the absence of severe glycemic impairment [36]. Furthermore, we observed that this risk disparity exhibited pronounced heterogeneity across gender and BMI subgroups, a pattern consistent with the sex-dependent fat distribution well-documented in recent epidemiological cohorts [32,37].

The substantially higher risk associated with Group 3 compared to Group 4 underscores a fundamental competition between urate overproduction and underexcretion. The TyG index primarily reflects IR-induced compensatory hyperinsulinemia [38], which upregulates the expression and activity of urate transporter 1 (URAT1) via the AKT signaling pathway, thereby exacerbating renal urate reabsorption [39]. However, our discordant phenotype data strongly imply that without a continuous, massive supply of urate, excretion impairment alone is insufficient to explain extreme SUA elevations. Visceral adiposity, represented by LAP, provides the essential metabolic substrate. Visceral fat is a highly active endocrine organ that releases excessive free fatty acids (FFAs) into the liver portal system. One potential mechanism involves specific molecular pathways. Preclinical models suggest that these FFAs may undergo β- oxidation, potentially activating HIF-1α and subsequently upregulating NT5C2 and xanthine dehydrogenase (XDH). This enzymatic cascade could drastically accelerate the de novo synthesis of uric acid from hypoxanthine [40,41]. Additionally, adipocytes can directly secrete purine substrates to further feed this enzymatic cascade [42]. This dual “catalyst and substrate” overproduction mechanism solidifies LAP as the paramount correlate of HUA. Subgroup analysis revealed distinct demographic differences, specifically a weakened association between the TyG index and HUA in males and individuals with a BMI ≥ 24 kg/m² without visceral adiposity. This finding can be explained by renal physiology. In the absence of visceral adiposity, extreme TyG elevation may reflect severe pancreatic β-cell dysfunction and uncontrolled hyperglycemia. When blood glucose levels exceed the renal threshold, glycosuria occurs [43]. This phenomenon might be attributable to a compensatory mechanism at the renal tubular level. Previous experimental studies have indicated that high concentrations of luminal glucose can competitively occupy the GLUT9b (isoform 2) transporter on the apical membrane of the proximal tubule, potentially suppressing urate reabsorption and facilitating uric acid-glucose exchange to promote uricosuria [44–46]. We hypothesize that TyG might exhibit a bidirectional effect on urate metabolism: early hyperinsulinemia retains urate [6,47], whereas decompensated glycosuria could theoretically promote its excretion. However, without urinary data, this mechanism remains speculative. Furthermore, the paradoxical risk decline at extreme TyG levels may partially reflect a statistical artifact due to data sparsity. Conversely, the high LAP phenotype conferred the highest relative risk in females (OR: 2.45). It is well established that under normal physiological conditions, estrogen (estradiol) shields women by promoting urate excretion via ABCG2 transporters [33]. However, we speculate that the severe systemic lipotoxicity and micro-inflammation generated by profound visceral adiposity could counteract the uricosuric effects of estrogen, diminishing this protective mechanism.

The absence of a multiplicative interaction between LAP and TyG signifies that visceral lipotoxicity and glucose-insulin dysregulation act as independent, additive pathways. However, evaluating interaction solely on a multiplicative scale may obscure the absolute clinical burden. Our subsequent analysis on an additive scale revealed a highly significant positive additive interaction (synergism). This dual phenomenon indicates that while these two discordant metabolic indices operate independently at the mechanistic level, their concurrent presence in a patient creates a synergistic clinical hazard—accounting for a substantial excess risk that is greater than the sum of their individual effects, with 15.1% of the HUA risk in the dual-high group specifically attributable to this clinical synergy. From a clinical translation perspective, this independence highlights the need to reconsider strategies in HUA management. Currently, metabolic risks in individuals with a normal BMI are frequently overlooked due to the inherent limitations of BMI, which fails to differentiate fat distribution and obscures unrecognized visceral adiposity [48,49]. LAP acts as a critical screening tool to unmask high-risk individuals hidden within normal weight brackets, effectively overcoming these inherent diagnostic limitations of BMI [50,51]. Given that visceral adiposity emerges as the principal metabolic correlate of HUA, interventions heavily reliant solely on insulin sensitizers (targeting TyG) may yield suboptimal urate-lowering results, especially in males and individuals with a BMI ≥ 24 kg/m². Clinical strategies must prioritize visceral fat reduction. Pharmacological agents capable of simultaneously targeting visceral adiposity and promoting uricosuria, such as GLP-1 receptor agonists or SGLT2 inhibitors, should be prioritized over isolated glycemic control [52,53]. Notably, SGLT2 inhibitors pharmacologically replicate the aforementioned glycosuria-induced urate excretion, rendering them particularly advantageous in interrupting the pathological progression of HUA [54,55].

### Limitation

Several limitations must be acknowledged. First, the cross-sectional design precludes causal inferences. Second, reliance on surrogate markers (LAP and TyG) rather than direct imaging or endocrine measurements may introduce bias. Third, although we strictly excluded individuals with a documented history of chronic kidney disease and those taking specific metabolic or urate-lowering medications, unmeasured confounding remains. Specifically, our dataset lacked continuous renal function metrics (e.g., eGFR) for multivariable adjustment and specific mechanistic biomarkers (e.g., urinary glucose and uric acid excretion fractions), as well as detailed questionnaire data on dietary habits (e.g., purine, alcohol, and fructose intake), physical activity, socioeconomic status, and the use of other potential uric acid-altering drugs (e.g., diuretics). Finally, validation in diverse ethnicities is needed, as our cohort was exclusively Chinese, and the median-based cutoffs utilized to define the metabolic phenotypes are inherently sample-dependent. Furthermore, by strictly excluding individuals taking relevant medications, our findings are most applicable to generally healthy or treatment-naive populations, which further limits the direct extrapolation of our results to high-risk clinical groups.

## Conclusions

In conclusion, visceral adiposity (evaluated by LAP) and insulin resistance (evaluated by the TyG index) are independently and additively associated with hyperuricemia, with visceral adiposity acting as the primary contributor. In the absence of concurrent visceral fat accumulation, isolated insulin resistance does not sustain high uric acid levels, potentially due to glycosuria-induced urate excretion. Furthermore, high visceral adiposity significantly attenuates the traditionally lower risk of hyperuricemia observed in females. Clinically, these findings highlight the utility of LAP over BMI for risk stratification and suggest that therapeutic strategies targeting visceral fat reduction and urate management should be prioritized over isolated glycemic control.

## Supporting information

S1 Table Sensitivity analysis of the association between metabolic phenotypes and HUA risk after excluding extreme demographic outliers (age ≥ 80 years or BMI ≥ 35 kg/m²). Model was adjusted for gender, age, smoking, drinking, systolic blood pressure (SBP), diastolic blood pressure (DBP), high-density lipoprotein cholesterol (HDL-C), and low-density lipoprotein cholesterol (LDL-C). A total of 703 individuals were excluded in this analysis.
(DOCX)
