# Supplementary material for: Independent and joint associations of visceral adiposity and insulin resistance with hyperuricemia: A large-scale cross-sectional study
Source: PLoS One. 2026 Sep 22;21(9):e0357105. doi: 10.1371/journal.pone.0357105 (PMC13596823; doi:10.1371/journal.pone.0357105)
Supplement: Supplementary file 1 [file pone.0357105.s001.docx]

Supplementary Table S1. Sensitivity analysis of the association between metabolic phenotypes and HUA risk after excluding extreme demographic outliers (age ≥ 80 years or BMI ≥ 35 kg/m²).

| **Phenotype** | **OR (95% CI)** | **P value** |
| --- | --- | --- |
| **Group 1 (Double-low)** | 1.00 (Reference) | — |
| **Group 2 (Double-high)** | 2.73 (2.59–2.88) | < 0.001 |
| **Group 3 (Isolated high LAP)** | 2.05 (1.90–2.21) | < 0.001 |
| **Group 4 (Isolated high TyG)** | 1.28 (1.17–1.39) | < 0.001 |

Model was adjusted for gender, age, smoking, drinking, systolic blood pressure (SBP), and diastolic blood pressure (DBP), high-density lipoprotein cholesterol (HDL-C), and low-density lipoprotein cholesterol (LDL-C). A total of 703 individuals were excluded in this analysis.
